# Useful vertical latissimus dorsi flap for partial breast reconstruction in every tumor location

**DOI:** 10.1186/s12893-022-01741-6

**Published:** 2022-07-28

**Authors:** Jong Ho Lee, Jeong Yeop Ryu, Kang Young Choi, Jung Dug Yang, Ho Yun Chung, Byung Chae Cho, Byungju Kang, Jeeyeon Lee, Ho Yong Park, Joon Seok Lee

**Affiliations:** 1 Department of Plastic and Reconstructive Surgery, School of Medicine, Kyungpook National University, Daegu, Republic of Korea; 2 Department of Surgery, School of Medicine, Kyungpook National University, kyungpook National University Chilgok Hospital, Daegu, 41404 Republic of Korea

**Keywords:** Vertial latissimus dorsi flap, Mini latissimus dorsi flap, Partial mastectomy, Partial breast reconstruction

## Abstract

**Background:**

We conducted a prospective cohort study to evaluate effective techniques for breast reconstruction after partial mastectomy due to breast cancer. Determining the method of reconstruction is often difficult as it depends on the location of the cancer and the amount of tissue excised.. Here, we present a new technique, using the vertical latissimus dorsi (LD) flap, that can be used in all partial mastectomies and can almost conceal scarring. We also compared these results to those of the mini LD flap.

**Methods:**

We analyzed the data of a total of 50 and 47 patients, who underwent breast reconstruction with the mini LD flap and the vertical LD flap, respectively. Immediately after tumor excision, breast reconstruction was initiated. The skin flap for vertical LD was designed in a planarian shape, such that it may be hidden as much as possible and minimize bulging during closure, and the LD muscle flap was designed with a sufficient distance in the inferior direction.

**Results:**

Our finding showed that the vertical LD flap group required significantly less total operation time than the mini LD flap group. While the mini-LD flap resulted in a scar that was difficult to conceal, the donor site scar of the vertical LD flap could not be seen easily, and no scar was visible on the back.

**Conclusions:**

The vertical LD flap is useful for partial breast reconstruction, in all breast regions requires a rather small volume of the flap. Moreover, recovery was relatively fast with high patient satisfaction.

**Supplementary Information:**

The online version contains supplementary material available at 10.1186/s12893-022-01741-6.

## Background

Breast cancer is the leading cause of cancer-related death in women and the most common cancer, with 2.3 million new cases diagnosed in 2020 [1] In women, breast cancer accounts for approximately 24.5% of all cancer cases and 15.5% of cancer-related deaths, ranking first in incidence and mortality in most countries in 2020 [2]. Patients with early-stage breast cancer is usually undergo surgery, including partial mastectomy with radiotherapy and breast-conserving surgery with radiotherapy if the invasion is less than 50% and 20% of the total breast volume, respectively [3]. Combined treatment is increasingly performed, as the oncologic safety based on the 5-year survival rate is similar to that of mastectomy alone [4–7]. If oncologic safety is confirmed, volume displacement reconstruction is conducted via oncoplastic breast surgery, which is appropriate when a medially located breast defect cannotbe reconstructed using the surrounding tissues, and when the defect size is > 3 cm [8, 9]. Not performing immediate partial breast reconstruction may lead to unsatisfactory aesthetic outcomes, such as breast distortion or retraction, noticeable volume changes, and secondary shape or position change, of the nipple-areolar complex [10].

**Fig. 1:**
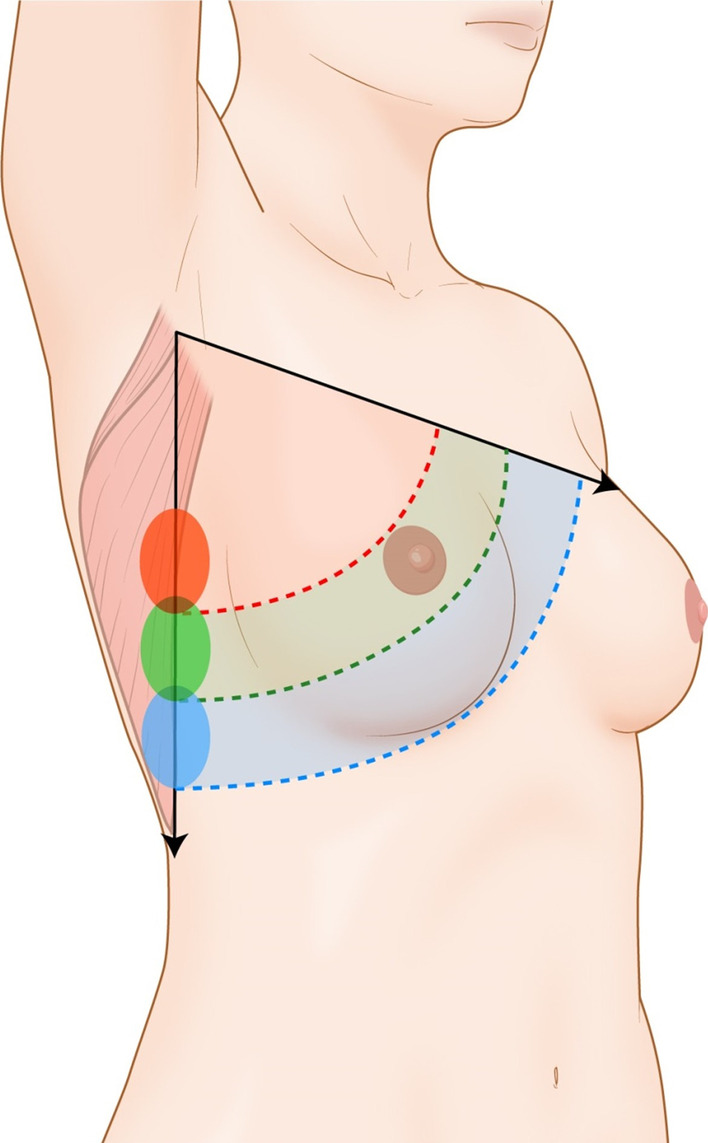
Concept illustration of the vertical latissimus dorsi (LD) flap. Vertical LD flaps cover most defects after partial mastectomy with a flap design on the mid-axillary line, according to the location of the defect. This is accomplished by setting the initiation of the thoracodorsal artery pedicle as a pivot point

The nature of the perforator flap used varies, depending on tumor location, preoperative breast volume, and the ratio of the excised mass.. The use of thoracodorsal artery perforator (TDAP) flap, lateral intercostal anterior perforator (LICAP) flap, anterior intercostal artery perforator (AICAP) flap, omental flap, mini latissimus dorsi (LD) flap modified from the classical LD flap, or muscle-sparing LD flap has been previously reported [11–16]. The mini LD flap, which has a similar design and technique to an extended LD flap, is designed as small as necessary for post-partial mastectomy defects in the cutaneous flap and dissected lesser in the inferior pole of the LD muscle. This is followed by transfer of a smaller volume of the LD flap for reconstruction, compared with the extended LD flap. However, reconstructive breast surgeons face challenges in determining the optimal surgical method when there is a defect in the lower medial portion, close to the midline of the body; it can be difficult to design the incision location of the mini LD flap such that it is hidden by the mid-axillary line. There are reports of customized reconstruction methods according to the location of the breast cancer and the ratio of excision amount to the breast size, with different rationales. However, because of differences in the surgical skills of reconstructive surgeons, and mere summaries of surgical techniques from previoues studies, consensus is yet to be established. Herein, we introduce a useful technique with a vertical LD flap on the mid-axillary line that can be used in all types of partial breast reconstructions, which largely conceals the incision area of the donor site (Fig. 1).

## Methods

### Study design

This study was conducted in accordance with the principles of the Declaration of Helsinki (revised in 2013). The Kyungpook National University Chilgok Hospital Institutional Review Board (No. 2019-12-006-002) approved this prospective study, and all patients provided informed consent for their data (including de-identified photographs) to be recorded, analyzed, and published for research purposes.

The data of a total of 50 and 47 patients who underwent immediate breast reconstruction with mini LD and vertical LD flaps, respectively, after partial mastectomy from September 2018 to June 2021, were analyzed.The patient of whom excised breast mass was under one third of the total preoperative breast volume was enrolled the vertical LD flap group instead of conventional mini LD flap. Recorded patient characteristics included age, BMI, any preoperative and postoperative appointment notes, postoperative 1 year breast volume assessed using the imaging software Volpara™ (version 1.5, Volpara Health Technologies), excised specimen weight, cancer staging, radiotherapy, chemotherapy, flap weight, total operative (op) time, flap elevation time, duration of indweeling negative-pressure, and total drain volume. Patient photometry follow-up was conducted preoperatively, intraoperatively, and at postoperative months (POM) 1, 3, and 6, and postoperative year (POY) 1. The total operation time was defined as the time from patient entry to exit from the operating room, registered in their electronic medical record. The reason why we calculated the operation time from the patient entry to exit on Table 1 was to highlight the vertical LD flap technique’s short operation time derived from immediate flap elevation after mass excision without waiting for the result of frozen biopsy or changing the patient’s position. Flap elevation time was calculated from the time flap incision design was completed, to the time when elevated flap was photographed right after the elevation was finished.. Patient satisfaction was evaluated using a pre-discharge satisfaction questionnaire, modified from the breast-Q questionnaire, consisting of seven detailed questions about five items. The questionnaire was evaluated using four scores: very satisfied, somewhat satisfied, somewhat dissatisfied, and very dissatisfied. Major complications, such as hematoma, seroma, flap loss, necrosis, and minor complications, including dehiscence, swelling, and sloughing, were recorded.

**Table 1:** Patient demographics

	Mini LD	Vertical LD	
No. of patients	50	47	
Factors	Value (%)	Value (%)	P value
Age (years)	45.4 ± 6.8	44.3 ± 13.1	0.1617
BMI (kg/m^2^)	22.5 ± 2.7	22.8 ± 3.2	0.8466
Diagnosis			
IDC	37 (76.0%)	27(57.4%)	
DCIS	9 (18.0%)	16(34.0%)	
ILC	1 (4.0%)	0(0%)	
Mucinous carcinoma	1 (2.0%)	2(4.3%)	
Phyllodes tumor	0 (0%)	2(4.3%)	
Breast volume	395.6 cc (± 160.1)	368.85 cc(± 97.6)	0.1019
Specimen weight	90.9(± 61.0)	71.4(± 54.1)	< 0.001
	151.7(± 75.9)	120.5(± 57.4)	
Flap weight	46 (96%)	30(100%)	< 0.001
Axillar surgery	2 (4%)	0(0%)	
SLNB	338.4 ± 49.5	256.0 ± 60.2	
AS	82.1 ± 41.4	61.8 ± 14.0	
Total OP time	13.1 ± 5.9	11.6 ± 2.3	< 0.001
Flap elevation time	11.1 ± 2.1	9.2 ± 2.3	0.0021
Admission duration	716.4 ± 306.4	524.4 ± 184.1	0.1019
Drain indwelling duration			< 0.001
Drain total volume			< 0.001

### Patient selection

This study was a prospective cohort study. Patient inclusion criteria were as follows: breast cancer patients between the ages of 30 and 60 years who underwent unilateral partial mastectomy andexcised approximately under one-third of their total breast volume. First of all, we have set the plan estimating the range of invasion on MRI evaluated by breast surgeon. On the next step, as soon as the excised mass is weighed under one third of the preoperative breast volume, we performed vertical LD flap instead of mini LD flap. The breast tissue density, as is known, has density close to 1, we identified the volume with weight.The following patients were excluded from the study: (I) patients with advanced breast cancer (stages 3 and 4), (II) patients with a history of cognitive impairment who could not complete the self-report questionnaire, (III) patients with history of any neurologic or musculoskeletal disorder, and (IV) patients with a history of alcohol or drug abuse. Patients with medially located breast cancer, far from the axillary pivot point, were informed that scar formation at the entire donor site may not be covered by a brassiere (bra). Patients were also informed that reconstruction using a mini LD flap could be conducted if the defect size was greatly increased after a frozen biopsy. Written consent was obtained from the patients prior to enrollment.

### Statistical analysis

Statistical analysis was conducted using regression analysis and t-test with SPSS (SPSS IBM 22.0). Correlations between op time, flap elevation time, admission duration, duration of indwelling drain and total drain volume according to both techniques, age, BMI, and breast preoperative volume were analyzed by regression analysis and comparative analysis between two cohort group’s satisfactory score by t-test.

### Preoperative design of mini LD flap and vertical LD flap

**Fig. 2:**
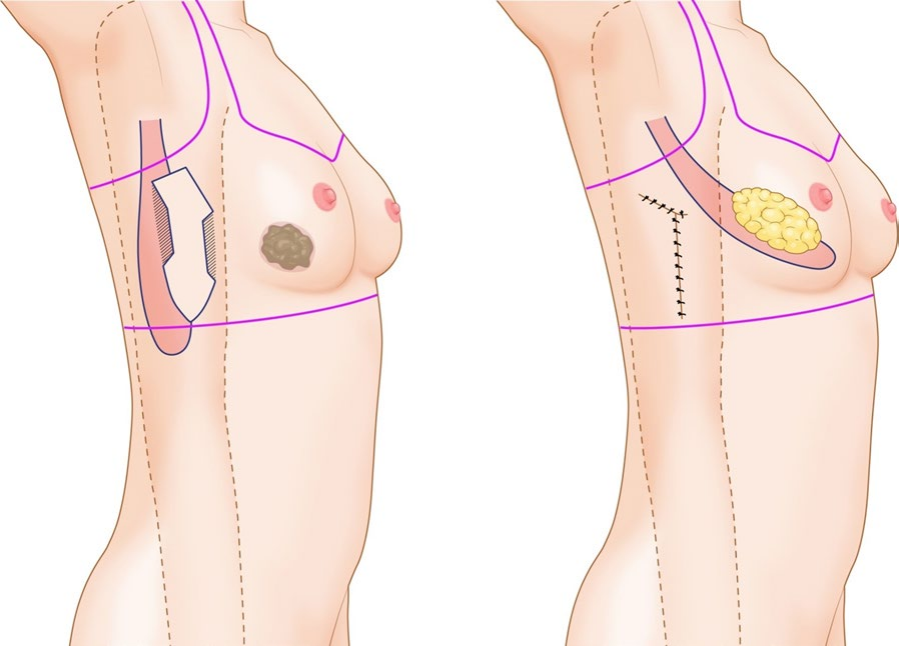
A vertical latissimus dorsi flap design for hidden scar formation. The classical planaria shape design enables linear scarring; unfortunately, it can cause severe dog ear and subsequent bulging on the axillary area with poor body contours on the inferior flank area. The newly devised planaria-shaped design incision enables better body contour and has the ability to hide the scar behind underwear. The size of the design is altered depending on the defect

The patient was asked to stand in an upright position while wearing a bra and the ipsilateral margin was marked. After removing the bra, the location of the breast cancer was marked and the distance measured from the axillary region. The transverse elliptical shaped skin flap of the mini LD flap was designed on the inner area of the marked bra line to hide the scar while the area covered by the arm was marked on the ipsilateral lateral side of the body for vertical LD flap in the attention position. The mini LD flap is designed in a typical trapezoid shaped fashion to include both transverse lateral branch and long vertical descending branch of thoracodorsal artery while the vertical LD flap that passed through the midline was designed to be at the same distance from the axilla as the cancer. The skin flap of the mini LD flap was designed in a planarian shape, such that it could be hidden by a bra as much as possible, and the LD muscle flap was designed with sufficient distance in the inferior direction (Fig. 2). The planarian head was designed to minimize axillary bulging during the closure.

### Mini LD flap technique for partial breast reconstruction

The operation was performed preceded by changing the patient position into lateral decubitous position after breast surgeon excised the mass. The patients were placed in a lateral decubitus position with an arm on the same side bent to 90, which was fixed on the stand. Skin paddle of the of the latissimus dorsi flap was desinged in small size transverse elliptical shape (approximately under 4cm x 12cm) allowing for easy closure of the donor site and for hiding of the scar in the bra. The local aneshtetic injection is followed by musculocutaneous LD flap election; then, the patient was turned to the supine position and the elevated flap with the attached skin paddle was rotated through the dissected axillar tunnel to the anterior chest wall area. The donor site was closed primarily over the flap and drains in the usual fashion, The donor area was closed in two layers using 2-0 Vicryl (Ethicon, Inc., Somerville, N.J.) simple stitches to the fascia and 4-0 Vicryl (Ethicon) for the superficial dermis. The exact skin defect was outlined on the transferred skin paddle, and the rest was de-epithelized. The mini-LD flap was fixed to recover the bulk of the breast and partially deepithelialized latissimus dorsi skin paddle was secured to the lower edges of the breast pocket by using 2-0 Vicryl (Ethicon) considering the symmetric breast contour.

### Vertical LD flap technique for partial breast reconstruction (video clip)

**Fig. 3:**
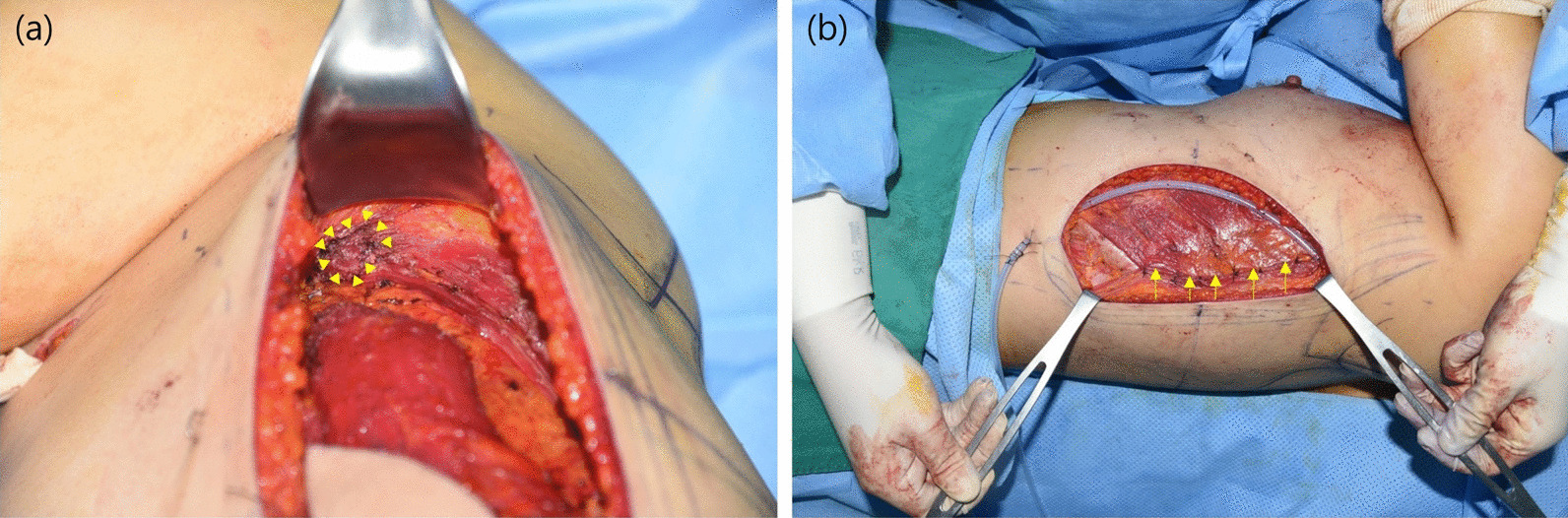
Intraoperative findings in the vertical latissimus dorsi (LD) flap technique. **a** Humeral detachment area after transferring the vertical LD flap through axillary tunneling. Detaching only 10 ~ 30% of the humeral insertion (yellow arrow head) enables the transfer. **b** Primary closure of the LD flap remnant after transfer. This technique maximizes postoperative functional recovery by restoring the remnant LD flap to the usual anatomical position (yellow arrow)

The drape was carefully placed to maintain the preoperative design. The cushion was placed on the ipsilateral back for easier tumor excision and axillary lymph node (LN) sampling by the breast surgeon. In addition, the vertical LD flap was designed to be open, without changing its position. First, the vertical LD flap procedure was started immediately after completing partial mastectomy and sentinel LN biopsy. Approximately 10–15 ccs of 1% lidocaine, mixed with epinephrine, was injected at the subcutaneous level, and the incision was made using a 10-blade to the preoperative design. The flap was elevated using a Bovie device (Bovie Medical Corporation, Clearwater, Florida, USA) which is a surgical device used to incise tissue, and to control bleeding by causing the coagulation of blood As the skin flap was small, the anterior border was preserved by tagging sutures of the skin and the LD muscle to prevent damage to the small perforators between them. Subsequently, the thoracodorsal artery pedicle was located by undermining the axillary direction, and Doppler ultrasound was perforemed to mark the pedicle artery in the direction of the descending branch. With reference to the macroscopically visible pedicle, the LD flap was marked and descended downward; the route that entered the muscle was marked as thoroughly as possible using Doppler ultrasound. Dissection was performed by undermining the inferior direction. The flap was cut from the inferior area through the superior area, considering flap inclusion of the pedicle, using the LigaSure device (LigaSure™, Minneapolis, MN, USA). The flap was elevated to a sufficient length and volume. After identifying the subscapular artery branching from the brachial artery, the pedicle was preserved around the area, and humoral insertion of the LD muscle was inferiorly dissected with minimal detachment. The main pedicle was macroscopically observed with the naked eye, and the thoracodorsal nerve was cut to allow for smoother movement of the flap. Once pinpoint bleeding was observed at the distal margin of the elevated vertical LD flap, the flap was tunneled and transferred through the axillary LN biopsy area to the breast defect area. The pedicle flap may bulge at the axillary position during tunneling; when severe bulging was observed, normal breast tissue along the route was released through scoring incision to allow the flap to pass through the tunnel. Skin tissue transfer was conducted only when simultaneous nipple reconstruction was needed. When the nipple was spared, the skin of the vertical LD flap was de-epithelized for breast reconstruction, and fixation suture was performed using 2–0 vicryl at three positions only on the upper margin of the flap, located on the defect. Then, two negative 400 cc drains were applied to the upper and lower margins of the flap. The donor site was restored to the original position of the vertical LD flap by mining the new anterior border of the LD muscle, and sutured using 2–0 vicryl. Next, the patient was placed on the surgical table in a sitting position, and the bra line and arm aligned to appear as a curved line, as much as possible. One 400 cc negative drain was placed, and 4 cc of fibrin glue was evenly sprayed. The operation was completed after layer-by-layer suturing (Fig. 3).

## Results

A total of 97 patients, including 50 who underwent immediate breast construction with a mini LD flap and 47 who underwent breast construction with vertical LD flaps, after partial mastectomy, were included during the period from September 2018 to June 2021. The mean age of the patients in the mini LD flap and vertical LD flap groups was 45.4 (± 6.8) and 44.3 (± 13.1) years, respectively. BMI was 22.5 (± 2.7) kg/m2 in the mini LD flap group and 22.8 (± 3.2) kg/m2 in the vertical LD flap group. Additionally, 37 (76%), 9 (18%), 1 (4%), 1 (2%), and 0 patients in the mini LD flap group were diagnosed with IDC, DCIS, ILC, mucinous carcinoma, and phyllodes tumor, respectively, whereas 27 (57.4%), 16 (34%), 0, 3 (4.3%), and 2 (4.3%) patients in the vertical LD flap group were diagnosed with IDC, DCIS, ILC, mucinous carcinoma, and Phyllodes tumor, respectively. The absolute values of the volume differences between the affected and unaffected sides were 21.57, 72.85, and 37.63 ccs in the vertical LD group and 26.61, 32.04, 7.63 ccs in the mini LD group preoperatively and at postoperative 6 months and 1 year, respectively. The excised specimen weight was 90.9 (± 61.0) g in the mini LD flap group and 71.4 (± 54.1) g in the vertical LD flap group. Moreoever, the flap weight was 151.7 (± 75.9) g and 120.5 (± 57.4) g in mini LD flap and vertical LD flap groups, respectively. For axillary surgery, sentinel lymph node biopsy (SLNB) was performed in 46 (96%) patients in the mini LD flap group and in 47 (100%) patients in the vertical LD flap group. Axillry lymph node biopsy (ALND) was performed in 2 (4%) patients in the mini LD flap group and in 0 patients in the vertical LD flap group. The total mean op time was 338 min in the mini LD flap group and 256 min in the vertical LD flap group. Flap elevation time was 82.1 min in the mini LD flap group and 61.8 min in the vertical LD flap group. The mean admission duration was 13.1 and 11.6 days in the mini LD flap and vertical LD flap groups, respectively. The mean donor site drain indwelling duration was 11.1 days in the mini LD flap group and 9.2 days in the vertical LD flap group. which underwent drain removal according to strict criteria when subsequent daily drain measurements were under 15 cc, to prevent recurrent seroma after drain removal. The total drain volume was 716 cc and 524 cc in the mini LD flap and vertical LD flap groups, respectively (Table 1). In the vertical LD flap group, mass weight, flap weight, op time, flap elevation timenegative pressure drain (Hemovac; Zimmer, Warsaw, Indiana, USA) duration, and H-V total volume were statistically significant (p < 0.05). However, there was no significant difference in terms of age. BMI significantly correlated with mass weight, flap weight, H-V duration, and total H-V drain volume. Preoperative breast volume was significantly correlated with mass weight, flap weight, flap elevation time, H-V duration, and H-V drain volume (Table 2). Patient satisfaction was as follows: breast symmetry scores of 3.2 ± 0.2 points for the mini LD flap group and 3.5 ± 0.4 points for the vertical LD flap group; unclothed breast shape scores of 3.1 ± 0.2 and 3.1 ± 0.3 the corresponding groups were recorded. Breast shapes on clothing scores were 3.6 ± 0.3 points in the mini LD flap group and 3.8 ± 0.1 in the vertical LD flap group; postoperative pain scores of 3.5 ± 0.1 points in the mini LD flap group and 3.7 ± 0.2 points in vertical LD flap group were reported. The scores for donor site scars were 3.7 ± 0.5 points in the mini LD flap group and 3.8 ± 0.4 points in the vertical LD flap group. Overall outcome scores were 3.7 ± 0.2 points in the mini LD flap group and 3.8 ± 0.1 points in the vertical LD flap group. Scores of 3.5 ± 0.1 points in the mini LD flap group and 3.8 ± 0.4 points in the vertical LD flap group were given for the item “Would you decide to undergo reconstruction with this operation method if you could go back to the earlier surgery? “ Statistical analysis on breast symmetry, clothed breast shape, donor site scar and overall outcome had statistical significance (Table 3).

**Table 2:** Statistical analysis using linear regression test

	Mass weight	Flap weight	OP time	Flap elevation time	Admission duration	Drain indwelling duration	Drain total volume
Group	0.002**	< 0.001**	< 0.001**	0.005**	0.135	< 0.001**	0.001**
age	0.832	0.786	0.445	0.297	0.533	0.435	0.599
BMI	0.002**	0.007**	0.195	0.471	0.128	0.010*	0.017*
breast volume	< 0.001**	0.004**	0.598	0.007**	0.166	0.040*	0.016*

** < 0.01, * < 0.05, *BMI* BODY MASS INDEX, *OP* OPERATION

**Table 3:** Patient satisfaction

Questionnaire	Very Satisfied (4)- Somewhat satisfied (3) Somewhat dissatisfied (2) Very dissatisfied (1)		
	Mini LD flap	Vertical LD flap	P-value
Breast symmetry			
How would you rate the symmetry of your reconstructed breast compared with contralateral side?	3.2 ± 0.2	3.5 ± 0.4	0.04
Breast shape			
How would you rate the shape of your unclothed reconstructed breast?	3.1 ± 0.2	3.1 ± 0.3	0.053
How natural is the shape of your reconstructed breast with clothing?	3.6 ± 0.3	3.8 ± 0.1	< 0.01
Postoperative pain			
How tolerable was the postoperative pain?	3.5 ± 0.1	3.7 ± 0.2	0.064
Donor site scar			
Considering its length and shape, how satisfied are you with your scar?	3.7 ± 0.5	3.8 ± 0.4	0.032
Overall outcome			
How satisfied are you with the overall outcome of the operation?	3.7 ± 0.2	3.8 ± 0.1	< 0.01
If you could go back before surgery, would you still decide to reconstruct with this method ?	3.5 ± 0.1	3.8 ± 0.4	0.025

The mean follow-up period was 1.31 years and 1.64 years in the vertical and mini LD groups, respectively. No major complications were observed after the operation, and the donor site seroma was resolved through bedside aspiration during the follow-up period within 3 weeks after discharge. Quilting sutures minimized dead space at the donor site ast they are suggested to reduce the required follow-up period for donor site seromas. Two cases each of seroma, and one case of linear necrotic changes at the suture in the mini LD and vertical LD groups, respectively, were resolved with simple dressings or additional revision operations.

## Discussion

The LD flap is a commonly used technique in reconstructive surgery. It is an autologous tissue that can cover a large area, for defects due to trauma, wide excision, or tumors. The LD flap was first reported in 1906, by Tansini and was used for the first time in breast reconstruction in 1970 [17–19]. It is one of the most commonly used flaps in reconstruction of small to moderate breast volumes [20]. In a recent study, the transfer of the unilateral LD muscle to the breast, with an extended LD flap, did not interfere with shoulder function or activities of daily living [21]. In case of insufficient flap volume, additionally, a single-center uncontrolled study also demonstrated Fat-Augmented LD (FALD) a safe and reliable technique. [22]. Mastectomy is a surgical treatment for breast cancer, which includes the aim of performing immediate breast reconstruction, simultaneously, after confirmation of oncologic safety. When patients have small breast volumes, total mastectomy, followed by reconstruction, is a viable option. However, in most cases, recent reports demonstrate equivalent oncologic safety in breast reconstruction after partial mastectomy and total mastectomy, with shorter operation times and better esthetic outcomes [23–25]. Therefore, to avoid radiotherapy, selection of total mastectomy does not ensure superiority in oncologic safety or esthetic outcome.

Various oncoplastic breast surgery techniques have been reported after partial mastectomy and breast-conserving surgery. Algorithms select reconstruction methods, such as TDAP, AICAP, LICAP, mini LD, and omental flap-based reconstruction, for the volume replacement technique, according to the size, shape, and location of breast cancer [26, 27]. However, as there are limitations, differences, and personal preferences in the training of reconstructive surgeons, the adequate use of all reported techniques is challenging. In addition, the use of a perforator flap requires an advanced surgical technique and may be difficult due to anatomical variation [28]. Therefore, this study reports a useful vertical LD technique that can more easily control the distance according to the defect by designing an LD flap pedicle of the thoracodorsal artery to the anterior border (mid-axillary region) compared with mini LD technique.

Our vertical LD technique provides easier access for the surgeon than the mini LD flap surgery.. It can properly reconstruct the breast, even if the location and range of the cancer change slightly.

. A previous study investigated the vascular pattern of the LD in cadavers [29]. To separate only the descending branch, more volume was required, and in some cases, the pedicle distance was sufficient [29]. While minimizing damage to the pedicle, the inferior part was cut to secure enough distance and the necessary muscles separated. Once the pedicle was visible with the naked eye, the flap was exfoliated while preserving the pedicle with minimal detachment of the uppermost humoral attachment area. This limits effects on shoulder function or limited range of motion. The flap was designed with a planarian head shape because an oval design of the flap may lead to the presence of remnants of bulging skin in the axillary area; as the LD muscle passes through, these remnants may seem more prominent. Therefore, the flap was designed with a planarian head shape with the uppermost vertex pulled down in the inferior direction, according to the principle of an advancement flap, thereby relieving any axillary bulge and concealing possible axillary scars behind the bra. Additionally, the lower part of the flap was designed to allow the linear scar of the donor site to maintain the S-line from the waist to the buttocks. As such, reconstruction can be conducted only through the incision of the LD muscle flap, without designing the skin flap. However, more muscle elevation is required to resolve the same volume of skin flap, and skin flap design is important, as atrophy may occur in future. Fat injection may be considered; however, the engraftment rate varies depending on the patient, and multiple re-operations may be necessary if insufficient amounts of fat are injected [30–32]. Furthermore, when the nipple or breast skin is excised due to involvement of cancer cells, simultaneous nipple reconstruction can be performed, and breast skin defects can be adequately covered [33].

In this study, the mini LD flap group that underwent partial breast reconstruction using only a portion of the LD flap and vertical LD flap group was used as two comparative cohort groups for comparison. Our findings showed that the vertical LD flap group required significantly less total operative time and flap elevation time than the mini LD flap group. This finding may be attributed to the following reasons: first, the surgery was conducted in the supine position without changing the patient's position, reducing the operation time. A vertical LD flap operation can be initiated without waiting for negative frozen biopsy results of the specimen. If cancer cells remain, additional excision can be performed by breast surgeons while prioritizing oncologic safety, and this can be compensated by further elevation of the vertical LD flap. Second, reconstruction with the vertical LD flap is easy to perform, as the long axis of the flap design coincides with the direction of the surgical field, whereas a classical mini LD flap may have the skin flap long axis perpendicular to the direction of the surgical approach. Thus, the operative field of view may be limited, affecting the total operation time. Finally, a vertical LD flap was used for smaller defects, which would have reduced the operation field size. The transverse long linear scar at the back, covered by the underwear, may not be visible to the patient; however, the surgical scar may be visible when the patient wears a swimsuit or summer clothing. In contrast, the donor site scar of the vertical LD flap could not be seen when the arm was lowered, and no scar was visible on the back. Moreover, restoration of the original muscle position by undermining the remnant LD muscle on the back side, after transfer of the vertical LD flap to the breast defect region, cannot be performed with the transverse design.

Breast volume analysis by Volpara™ readings showed a strong correlation with actual mastectomy volume measurements in a previous study [34]. The volume difference analysis between the affected and unaffected sides of the breast demonstrated fewer differences in the vertical LD group at the 1-year postoperative follow-up, although the difference was not statistically significant. These objective data are considered to correlate deeply with breast symmetry, which is one of the most important factors in breast surgery and requires further analysis (Table 1).

**Fig. 4:**
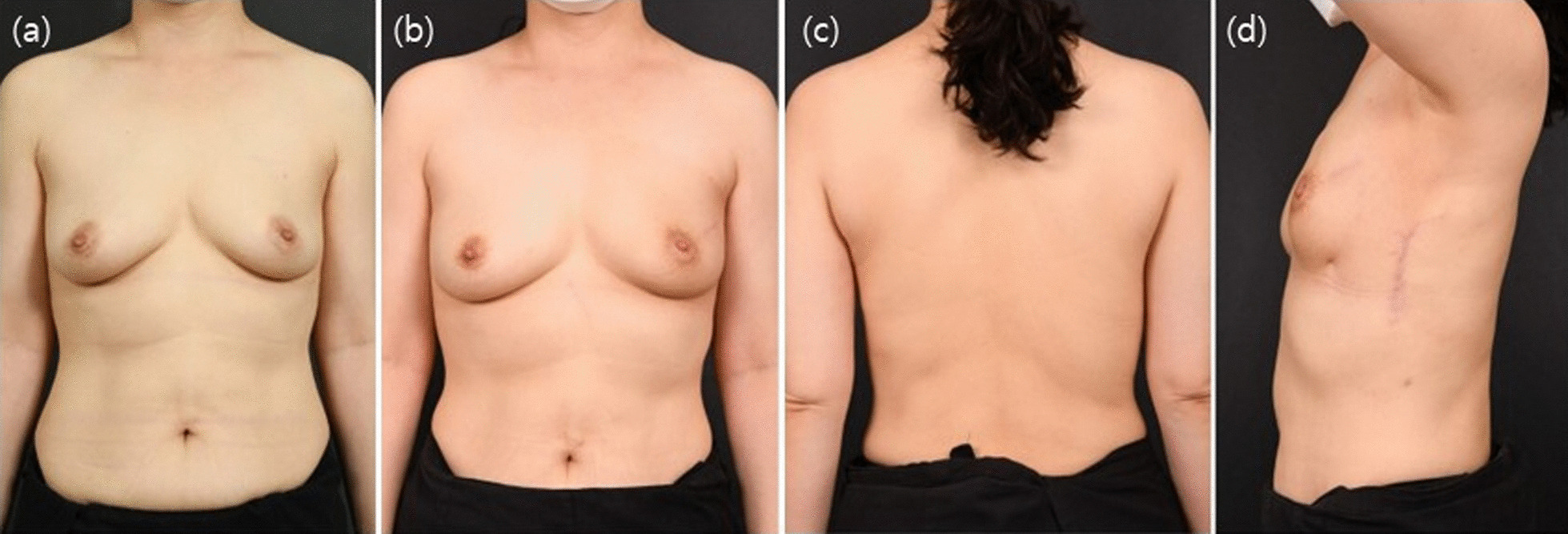
Breast cancer on the upper lateral area. **a** Preoperative findings; **b**–**d** postoperative 1-year findings: AP, PA, and lateral views. The mass on the upper lateral area is the most appropriate location for vertical latissimus dorsi (LD) flap reconstruction, as it is not required for a long pedicle and shows acceptable outcomes with low complications by transferring the LD flap directly through the axillary lymph node biopsy area. In addition, there is no visible scar on the back by use of this technique and a hidden scar on the donor site

**Fig. 5:**
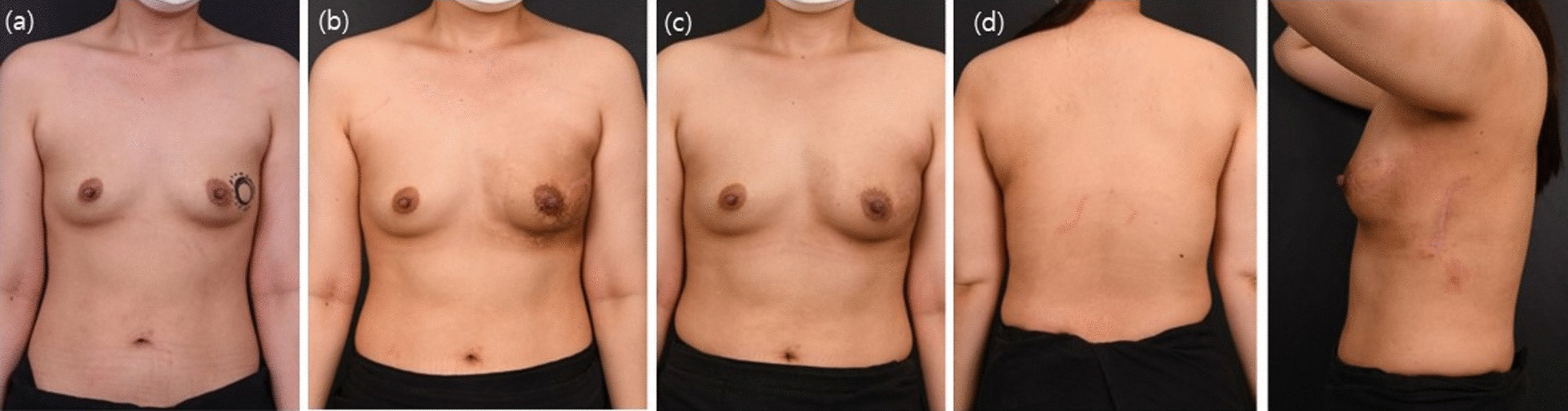
Breast cancer in the middle lateral area. In this patient, skin excision was performed in a circular shape because the cancer was in close proximity to the breast skin. The skin was then covered with a vertical latissimus dorsi (LD) flap. The patient’s progress after radiation therapy has been favorable. **a** Preoperative findings; **b** Postoperative 3-month findings, observed immediately after radiotherapy. Suitable esthetic outcomes are observed, except for the pigmentation caused by radiation. **c** Postoperative 6-month findings: AP view. The breast contour is well sustained despite radiotherapy, and the pigmentation has subsided. **d** Postoperative 6-month findings: PA and lateral views. No scar is visible onfrom the back

**Fig. 6:**
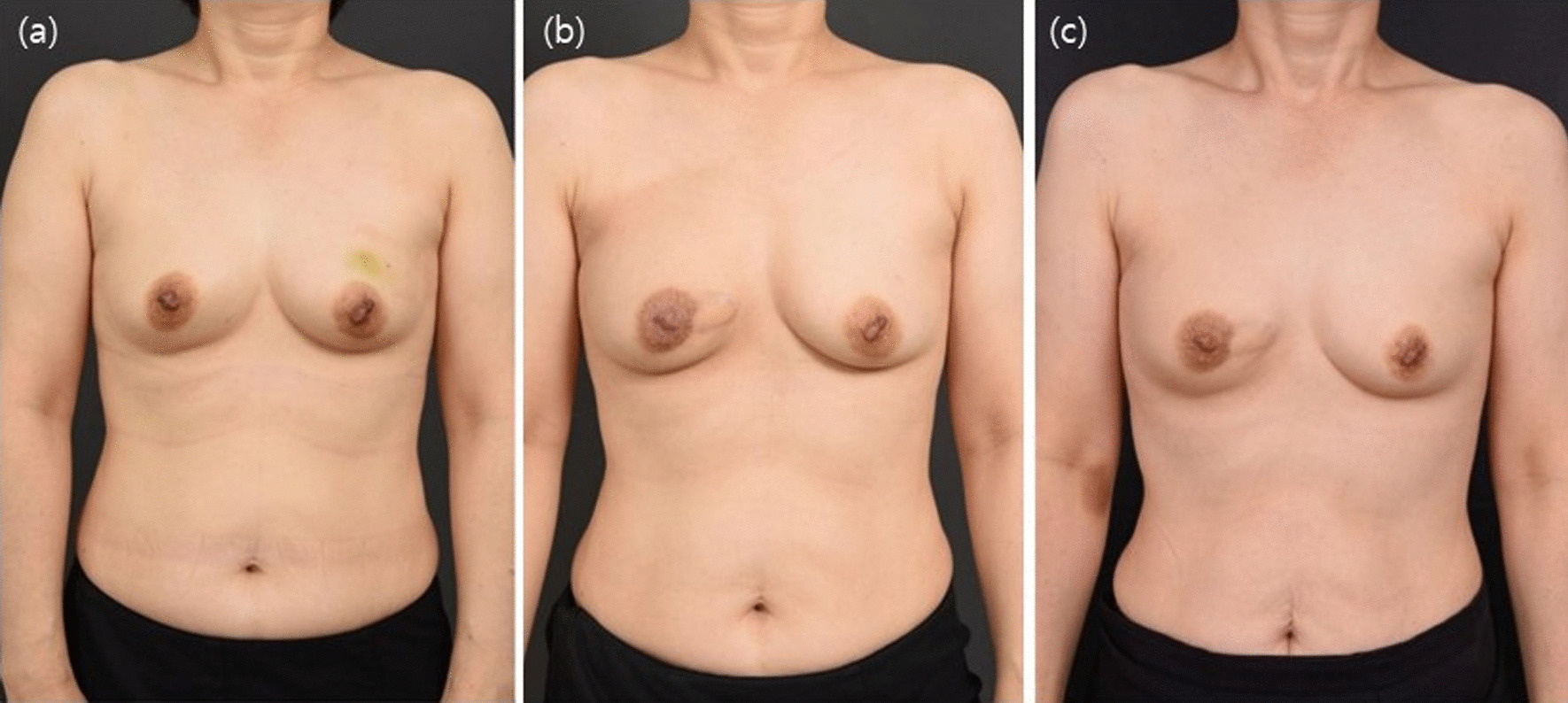
Breast cancer on the lower medial area. **a** preoperative findings; **b** postoperative 6-month findings; **c** postoperative 2-year findings. Long-term follow-up shows sustained stable coverage, even on the lower medial located defect, without any bulging on the course of the pedicle muscle or depression accompanied with volume reduction

**Fig. 7:**
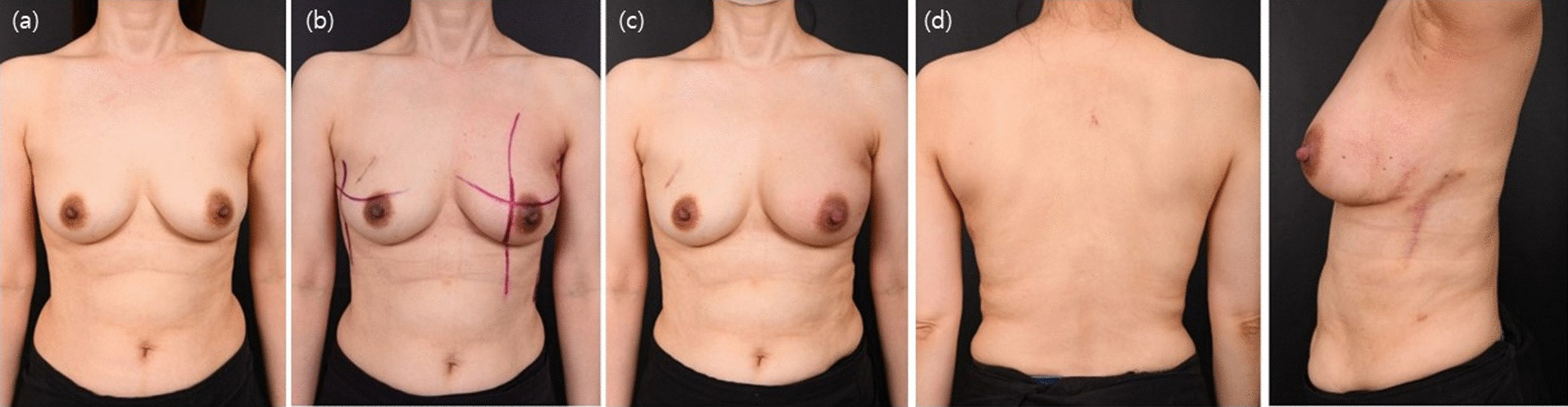
Breast cancer in the lower lateral area. In this patient, the cancer site could be accessed through the IMF. The patient has no scar on the breast mound; the incision scar is hidden by the IMF. Furthermore, the donor site scar is located in a region that can be covered by her bra and arm. **a** Preoperative findings; **b** Postoperative 3-month findings. Suitable esthetic outcomes are observed except for mild pigmentation caused by radiotherapy. **c** Postoperative 7-month findings: AP view. The breast contour is well sustained despite radiotherapy, and pigmentation has subsided. **d** Postoperative 7-month findings: PA and lateral views. No scar is visible on the back

**Fig. 8:**
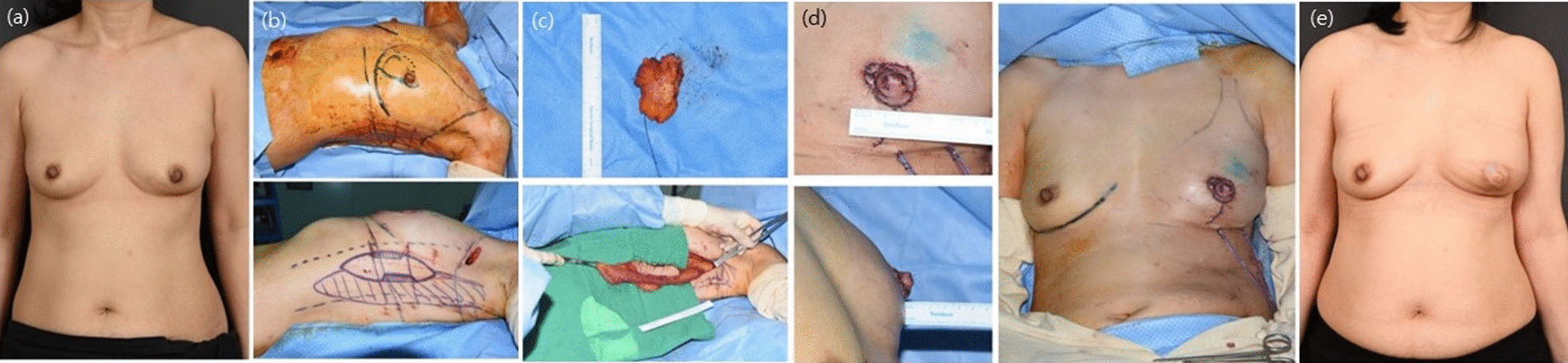
Simultaneous breast and nipple reconstruction findings using the vertical latissimus dorsi (LD) flap technique. **a** Preoperative findings; **b** Breast and vertical LD flap design photos; **c** Excised tumor and elevated vertical LD flap; **d** Immediate postoperative findings; **e** Postoperative 1-year findings. The vertical LD flap reconstruction is accompanied with simultaneous nipple reconstruction (SNR) by using a skin flap from the vertical LD to provide a suitable nipple outcome

However, these advantages do not lead to the complete replacement of partial reconstruction techniques, including the mini LD flap. If more than one-third of the breast volume is removed, a moderate volume of the flap is needed. In such cases, a mini LD flap is required. Patients must be fully informed about these possible intraoperative variations prior to surgery, to determine the most suitable surgical technique. Although a longitudinal extension of the vertical LD flap design may allow the use of an extended LD flap, a scar that is visible to the patient may be formed. In addition, the medial part can only be reached after changing patient position to the decubitus position during the operation. This eliminates the advantages of the vertical LD flap, and longitudinal extension of the vertical LD flap design is not considered an appropriate indication. Therefore, a vertical LD flap can reconstruct defects in all breast regions and is very useful as a replacement reconstruction technique that requires a rather small flap volume, especially in patients with a small breast volume. The statistical results showed relatively faster recovery and higher overall patient satisfaction with statistical significance ((Table 3). As such, a vertical LD flap is a pedicled flap among partial breast reconstruction techniques and has the same pedicle as an extended LD flap with better accessibility. A vertical LD flap may have positive effects on the algorithm of breast reconstruction using the LD flap, which allows reconstruction regardless of tumor location in the breast. Although less than one-third of the total breast volume was incised, a vertical LD flap may be an appropriate indication when the volume displacement technique is not feasible with the remaining tissues of small breast volumes if the nipple and the breast skin are excised. Herein, we report a vertical LD flap modified from an ordinary mini LD flap, which is a useful technique, capable of being used for all partial breast reconstructions. Small breast cancer in the upper lateral region is the best indication for the use of a vertical LD flap, and this technique allows excision of the excised breast skin (Figs. 4, 5). Reconstruction of defects in the lower medial parts, which requires the longest flap to reach the defect, also showed good long-term esthetic outcomes(Fig. 6). Partial mastectomy along the inframammary fold line, for defects of the lower lateral part, was also associated with good outcomes and no scars on the breast mound (Fig. 7). If the nipple is also involved, simultaneous nipple reconstruction can be performed using the skin flap of the vertical LD flap (Fig. 8). These advantages are considered reasons for superior patient psychological satisfaction (Table 3) and subsequent high patient satisfaction.

However, our study has the following limitations: first, as breast-conserving surgery becomes increasingly popular, long-term locoregional recurrence rates must be monitored, and the potential need for completion mastectomy and whole breast reconstruction (down the line in the event of a local recurrence) is a disadvantage of using an LD flap for partial breast reconstruction; however, our study only had a brief follow-up period. In the reconstruction of defects in the medial part of the breast, vertical flap designs may be slightly downward, such that the donor site may not be hidden under the bra, and as a result, the scar may be visible to the patient. Although a vertical LD flap is more applicable for defects regardless of their location, it has limitations in replacing the classic LD flap technique or a mini LD flap, in cases of moderate to large breast defects. Lastly, mild bulging is observed in the axillary area where the pedicle muscle has passed, although bulging is less than that with the mini LD flap or the extended LD flap techniques. Bulging is improved after six months in most cases; however, delicate procedures may be required to minimize muscles close to the pedicle. Furthermore, if the descending and transverse branches of the thoracodorsal artery diverge in the distal region, separation may not be possible with only the descending branch.

## Conclusions

The vertical LD flap is applicable in all partial breast reconstructions, regardless of the region of defect. It also showed a statistically significant decrease in admission and operation times with higher patient satisfaction than the mini LD flap technique in the present study. Although further evaluations are required, the mini LD flap with a vertical LD flap may be an alternative technique for future partial breast reconstructions.

## Supplementary Information

**Additional file 1. Video S1.** Vertical latissimus dorsi flap operative technique for partial breast reconstruction.

**Additional file 2.** Vertical LD video.

## Data Availability

All data generated or analysed during this study are included in this published article (Additional files 1 and 2).

## References

[1] Sung H, Ferlay J, Siegel RL (2021). Global cancer statistics 2020: GLOBOCAN estimates of incidence and mortality worldwide for 36 cancers in 185 countries. CA Cancer J Clin.

[2] Lei S, Zheng R, Zhang S (2021). Global patterns of breast cancer incidence and mortality: a population-based cancer registry data analysis from 2000 to 2020. Cancer Commun (Lond).

[3] Rainsbury RM, Paramanathan N (2007). UK survey of partial mastectomy and reconstruction. Breast.

[4] Veronesi U, Cascinelli N, Mariani L (2002). Twenty-year follow-up of a randomized study comparing breast-conserving surgery with radical mastectomy for early breast cancer. N Engl J Med.

[5] Fisher B, Anderson S, Bryant J (2002). Twenty-year follow-up of a randomized trial comparing total mastectomy, lumpectomy, and lumpectomy plus irradiation for the treatment of invasive breast cancer. N Engl J Med.

[6] Huston TL, Simmons RM (2005). Locally recurrent breast cancer after conservation therapy. Am J Surg.

[7] Lee J, Jung JH, Kim WW (2017). Five-year oncologic outcomes of volume displacement procedures after partial mastectomy for breast cancer. Clin Breast Cancer.

[8] Yang JD, Lee JW, Cho YK (2012). Surgical techniques for personalized oncoplastic surgery in breast cancer patients with small- to moderate-sized breasts (part 1): Volume displacement. J Breast Cancer.

[9] Yang JD, Kim MC, Lee JW (2012). Usefulness of oncoplastic volume replacement techniques after breast conserving surgery in small to moderate-sized breasts. Arch Plast Surg.

[10] Audretsch WP, Spear SL, Willey SC, Robb GL (2006). Reconstruction of the partial mastectomy defect: classification and method. Surgery of the breast: principles and art.

[11] Hamdi M, Van Landuyt K, Spear SL, Willey SC, Robb GL (2006). Pedicled perforator flaps. Surgery of the breast: principles and art.

[12] Masia J, Clavero JA, Larrañaga JR (2006). Multidetector-row computed tomography in the planning of abdominal perforator flaps. J Plast Reconstr Aesthet Surg.

[13] Hamdi M, Van Landuyt K, de Frene B (2006). The versatility of the inter-costal artery perforator (ICAP) flaps. J Plast Reconstr Aesthet Surg.

[14] Hamdi M, Wolfli J, Van Landuyt K (2007). Partial mastectomy reconstruction. Clin Plast Surg.

[15] Yang JD, Lee J, Lee JS (2021). Aesthetic scar-less mastectomy and breast reconstruction. J Breast Cancer.

[16] Elzawawy EM, Kelada MN, Al Karmouty AF (2018). Design of mini latissimus dorsi flap based on thoracodorsal vascular patterns. Ann Plast Surg.

[17] Ribuffo D, Cigna E, Gerald GL (2015). Iginio Tansini revisited. Eur Rev Med Pharmacol Sci.

[18] Olivari N (1976). The latissimus flap. Br J Plast Surg.

[19] Muhlbauer W, Olbrisch R (1977). The latissimus dorsi myocutaneous flap for breast reconstruction. Chir Plast.

[20] Chang DW, Youssef A, Cha S (2002). Autologous breast reconstruction with the extended latissimus dorsi flap. Plast Reconstr Surg.

[21] Lee JS, Park E, Lee JH (2021). Alteration in skeletal posture between breast reconstruction with latissimus dorsi flap and mastectomy: A prospective comparison study. Gland Surg.

[22] FS di Pompeo, G D'Orsi, G Firmani, et al. Total breast reconstruction with the fat-augmented latissimus dorsi (FALD) flap: high safety in a single-center uncontrolled case series. J Plast Reconstr Aesthet Surg. 2022 10.1016/j.bjps.2022.06.052 35907690

[23] Lee J, Jung JH, Kim WW (2015). Oncologic outcomes of volume replacement technique after partial mastectomy for breast cancer: a single center analysis. Surg Oncol.

[24] Lee J, Jung JH, Kim WW (2017). Five-year oncologic outcomes of volume displacement procedures after partial mastectomy for breast cancer. Clin Breast Cancer.

[25] Lee J, Jung JH, Kim WW (2018). Comparison of 5-year oncological outcomes of breast cancer based on surgery type. ANZ J Surg.

[26] Mericli AF, Szpalski C, Schaverien MV (2019). The latissimus dorsi myocutaneous flap is a safe and effective method of partial breast reconstruction in the setting of breast-conserving therapy. Plast Reconstr Surg.

[27] Youssif S, Hassan Y, Tohamy A (2019). Pedicled local flaps: a reliable reconstructive tool for partial breast defects. Gland Surg.

[28] Pignatti M, Tos P, Garusi C (2020). A sequence of flaps and dissection exercises in the living model to improve the learning curve for perforator flap surgery. Injury.

[29] Cai R, Xie Z, Zhou L (2018). Pedicled descending branch latissimus dorsi mini-flap for repairing partial mastectomy defect: a new technique. Plast Reconstr Surg Glob Open.

[30] Santanelli di Pompeo F, Laporta R, Sorotos M, Pagnoni M, Falesiedi F, Longo B. Latissimus dorsi flap for total autologous immediate breast reconstruction without implants. Plast Reconstr Surg 2014;134:871e-879e. 10.1097/PRS.0000000000000859 25415109

[31] Economides JM, Song DH (2018). Latissimus dorsi and immediate fat transfer (LIFT) for complete autologous breast reconstruction. Plast Reconstr Surg Glob Open.

[32] Demiri EC, Dionyssiou DD, Tsimponis A, Goula CO, Pavlidis LC, Spyropoulou GA (2018). Outcomes of fat-augmented latissimus dorsi (FALD) flap versus implant-based latissimus dorsi flap for delayed post-radiation breast reconstruction. Aesthetic Plast Surg.

[33] Lee JH, Ryu JY, Lee JH (2021). Simultaneous nipple reconstruction in autologous breast reconstruction. Gland Surg.

[34] Teo I, Whelehan P, Macaskill JE, Vinnicombe S, Munnoch DA, Evans A (2016). Volpara™ as a measurement tool for breast volume. J Plast Reconstr Aesthet Surg.

