# Supplementary material for: Useful vertical latissimus dorsi flap for partial breast reconstruction in every tumor location
Source: BMC Surg. 2022 Jul 28;22:294. doi: 10.1186/s12893-022-01741-6 (PMC9336010; doi:10.1186/s12893-022-01741-6)
Supplement: Supplementary file 1 — Additional file 1. Video S1. Vertical latissimus dorsi flap operative technique for partial breast reconstruction. [file 12893_2022_1741_MOESM1_ESM.docx]

**Supplementary Video Legend**

**Supplementary Video. Vertical latissimus dorsi flap operative technique for partial breast reconstruction**
